# Chamomile Tincture and Lidocaine Hydrochloride Gel Ameliorates Periodontitis: A Preclinical Study

**DOI:** 10.3390/biomedicines12112629

**Published:** 2024-11-17

**Authors:** Jiahui Sun, Huiyi Wang, Junhong Xiao, Qiudong Yang, Heyu Liu, Zhengkun Yang, Yuqi Liu, Xin Huang, Liu Yang, Li Ma, Zhengguo Cao

**Affiliations:** 1 State Key Laboratory of Oral & Maxillofacial Reconstruction and Regeneration, Key Laboratory of Oral Biomedicine Ministry of Education, Hubei Key Laboratory of Stomatology, School & Hospital of Stomatology, Wuhan University, Wuhan 430079, China; sunjiahui@whu.edu.cn (J.S.); 2014301040148@whu.edu.cn (H.W.); junhongxiao@whu.edu.cn (J.X.); 2022103040006@whu.edu.cn (Q.Y.); liuheyu21@whu.edu.cn (H.L.); 2017302220097@whu.edu.cn (Z.Y.); liuyuqi@whu.edu.cn (Y.L.); huangxin1994@whu.edu.cn (X.H.); leah_yangl@whu.edu.cn (L.Y.); 2 Department of Periodontology, School & Hospital of Stomatology, Wuhan University, Wuhan 430079, China

**Keywords:** periodontitis, chamomile, inflammation, Type II interferon (IFN-γ)

## Abstract

**Background/Objectives**: Periodontitis is a common oral disease marked by gingival inflammation and alveolar bone loss. This study evaluated the efficacy of chamomile tincture and lidocaine hydrochloride (CLH) gel in mitigating periodontal inflammation and bone loss and uncovered the molecular mechanisms involved, both in vitro and in vivo. **Methods**: A periodontitis model was induced in Sprague Dawley rats by ligating the mandibular first molars. Sixty rats were divided into four groups: control (C), periodontitis (PD), periodontitis + CLH gel once daily (G1), and periodontitis + CLH gel thrice daily (G3). Clinical, micro-computed tomography (micro-CT), biological, and histological evaluations were performed, focusing on osteoclastogenesis, osteogenesis, and inflammatory cytokine production. The effect of CLH gel on inflammatory responses in RAW264.7 cells was also assessed through co-culture assays under *Porphyromonas gingivalis* (*P. gingivalis*) infection, with RNA-sequencing, qPCR, and Western blot analyses to explore underlying mechanisms. **Results**: CLH gel significantly reduced gingival and systemic inflammation and mitigated bone loss by enhancing the bone volume to tissue volume ratio and trabecular thickness via the RANKL/OPG axis in rats. The G3 group showed marked reductions in osteoclasts and increases in osterix-positive cells compared to other groups. In vitro, CLH gel reduced the inflammatory phenotype of macrophages in the periodontitis microenvironment by modulating Type II interferon (IFN-γ) networks. **Conclusions**: CLH gel reduced inflammation and bone loss in rat periodontitis, promoting osteogenesis and inhibiting osteoclastogenesis. It also suppressed macrophage inflammation via Type II interferon networks under *P. gingivalis* stimulation. These findings suggest that CLH gel has potential as an adjunctive therapy for periodontitis.

## 1. Introduction

Periodontitis, a chronic inflammatory disease triggered by dental plaque, contributes to adult tooth loss, affecting general health and quality of life [1]. In the inflammatory microenvironment shaped by periodontopathogenic bacteria, macrophages are recruited and differentiate into the M1 phenotype. These M1 macrophages produce cytokines like IL-6, TNF-α, and IL-1β, activating other immune cells and accelerating alveolar bone destruction [2]. The primary goals in managing periodontal disease include reducing the bacterial load, inhibiting inflammation progression, and regenerating the impaired periodontal tissues [3]. Although clinical practices, such as scaling and root planing, partially impede disease progression, they fall short of sufficient plaque elimination and tissue regeneration [4]. Furthermore, the adjunctive therapies in use rely heavily on antibiotics, such as minocycline hydrochloride ointment, for biofilm control [5], resulting in drug resistance or other side effects like tooth discoloration, inhibition of bone formation, and hepatorenal toxicity [6,7].

Herbal extracts are known for their safety profile with fewer complications and are a crucial source of medicinal supply globally [8,9,10]. Recently, there has been a resurgent interest in using herbal remedies, such as baicalin [11], propolis [12], artemisinin [13], and Panax ginseng [14], for periodontal therapies. Chamomile tincture is a liquid extract from German chamomile flowers (*Matricaria recutita* L.). It has anti-inflammatory and antioxidant properties, rich in essential oils like α-bisabolol, chamazulene, sesquiterpene lactones, and flavonoids [15]. Listed as Generally Recognized as Safe (GRAS) by the US Food and Drug Administration [16], studies have shown that chamomile exhibits potential therapeutic effects on oral mucositis and gingival inflammation [17,18,19,20,21], owing to its sedative, anti-inflammatory, and antimicrobial properties.

Chamomile tincture and lidocaine hydrochloride gel (KAMISTAD ^®^ GEL) is a pre-packaged medication available on the market. Its primary ingredients consist of 200 mg of chamomile tincture and 20 mg of lidocaine hydrochloride per gram of gel. It has demonstrated intrinsic anti-inflammatory and anesthetic capacity with good adhesion for localized application. In a recent clinical study comprising human wisdom teeth with periodontitis, local smear plus irrigation with CLH gel significantly shortened the treatment time by relieving inflammation and pain [22]. The success of this approach, equally effective in mitigating patient discomfort and sensitivity during mechanical plaque removal in chronic periodontitis therapy, can be predominantly credited to the anesthetic properties of lidocaine. In addition, an observational study reported that CLH gel has similar effects to minocycline hydrochloride ointment in reducing the quantities of *Porphyromonas gingivalis* and *Prevotella intermedia* in subgingival plaque and improving the clinical parameters such as probing depth, bleeding index, and attachment loss [23]. Given the antimicrobial properties reported for this compound gel, the anesthetic component’s potential for enhancing treatment comfort, and the anti-inflammatory potential of its main ingredient, chamomile, this study was designed to explore its overall clinical efficacy.

These experiments aim to provide a solid theoretical basis for its potential use as an adjunct therapy in non-surgical treatment of periodontitis. We established a rat periodontitis model and a *P. gingivalis*-infected macrophage assay to evaluate the potential pharmacological effects of CLH gel in vitro and in vivo. The findings revealed its protective properties on the anti-inflammatory response and alveolar bone regeneration in periodontitis.

## 2. Materials and Methods

### 2.1. Gel Preparation and Animals

Chamomile tincture and lidocaine hydrochloride gels (KAMISTAD ^®^ GEL, catalog number: H20150426) were synthesized by STADA Consumer Health Deutschland GmbH (Bad Vilbel, Germany), containing chamomile tincture (200 mg/g) and lidocaine hydrochloride (20 mg/g) with density = 1.33 (g/cm^3^). This commercially available gel was directly applied in subsequent experiments without any modification.

All animal procedures were performed with the approval of the Ethics Committee of the Hospital of Stomatology, Wuhan University (S07922080L), and complied with the ethical standards and guidelines in the 8th edition of the Guide for the Care and Use of Laboratory Animals (National Research Council, Rockville, MD, USA, 2011). Sixty male Sprague Dawley rats (7–8 weeks old; 250–280 g) were bred in a specific pathogen-free environment under controlled light (12-h light/12-h dark schedule) and temperature (22 ± 2 °C), with the same diet and hydration conditions.

The rats were randomly divided into four groups: C (control group, no intervention), PD (induced with periodontitis), G1 (induced with periodontitis and treated with CLH gel once a day), and G3 (induced with periodontitis and treated with CLH gel thrice a day). After one week of acclimation, the rats in the experimental groups (PD, G1, G3) were weighed, and an intraperitoneal injection of pentobarbital sodium (1.5%, 30 mg/kg) was administered for anesthesia. Subsequently, 4–0 silk threads were placed and fixed around the cervical regions of the bilateral mandibular first molars as described previously [24,25]. The ligatures were monitored daily for signs of loosening and retied in case of any loosening. After 7 days of ligature application, 100 μL of CLH gel was injected into the sulcus along the periodontal pocket for seven consecutive days, once a day in the G1 group and three times a day in the G3 group. Drinking water was prohibited for one hour following gel administration. Considering previous observations that bone resorption ceases to worsen progressively during the chronic phase (14–21 days), our assessment focused on the histological, radiographic, and biochemical changes in the early stages of disease progression (14 days) [26]. All the animals were sacrificed by carbon dioxide asphyxiation after blood collection on the 14th day.

### 2.2. Sample Collection

Whole blood samples were collected from the abdominal aorta (n = 3/group), immediately centrifuged to obtain the serum, and stored at −80 °C until further analysis. The mandibles were dissected (n = 10/group), and gingival tissues surrounding the first molars were separated under a stereomicroscope and stored in RNAlater solution (Invitrogen, Carlsbad, CA, USA, Cat No. AM7021) (n = 3/group). The organs of rats (n = 6/group) (heart, liver, spleen, kidney, and lung) were removed for histopathological analysis. The mandibles and organs were fixed in 4% paraformaldehyde.

### 2.3. Clinical Attachment Loss (CAL) and Probing Pocket Depth (PPD)

The distance between the cemento-enamel junction (CEJ) and the coronal position of the junctional epithelium attached to the root surface (i.e., the CAL) was measured as described previously, taken from the CEJ to the coronal margin of the attached junctional epithelium on the root surface (CAL) [27]. The evaluation was performed using hematoxylin and eosin (H&E)-stained sections and image-analysis software. The PPD was evaluated at three points (mesial, middle, and distal) on the buccal side of the mandible’s first molar from the gingival margin to the bottom of the pocket (n = 10/group). It was measured using a periodontal probe (PCP UNC-15) under light pressure by the same person.

### 2.4. Micro-CT and Three-Dimensional (3D) Reconstruction of the Alveolar Bone

After fixation in 4% paraformaldehyde for 24 h, the separated mandibles were preserved in 75% alcohol for further use. They were scanned by a Quantum GX micro-CT scanner (PerkinElmer Inc., Waltham, MA, USA), operating at 100 kV, 200 μA, and 10 μm. The NRecon software 1.6.10.4 was used to indicate 3D reconstructions of the alveolar bones (n = 10/group), and DataViewer 1.5.4.0 was used for image repositioning and measurements of the distance between the CEJ and the alveolar bone crest (ABC), referred to as the CEJ-ABC. The bone volume to tissue volume ratio (BV/TV), trabecular thickness (Tb.Th), trabecular separation (Tb.Sp), and trabecular number (Tb.N) were measured using CT Analyser 1.15.4.0 and CTvol 2.2.3.0. The alveolar bone area under the furcation of mandibular first molars, from the mesial root to the distal root, was defined as the region of interest (ROI), as described previously [28].

### 2.5. Histochemical and Histomorphometric Microscopic Analysis

The mandibular tissue samples underwent decalcification with 10% EDTA (Servicebio, Wuhan, China, G1105-500ML), followed by a gradient alcohol dehydration process and overnight clarification in n-butanol. After paraffin embedding, the tissue blocks were mounted on a microtome, and 5-μm-thick sections were prepared for subsequent histological examinations (n = 4/group for each parameter).

#### 2.5.1. H&E and Masson’s Trichrome Staining

After deparaffinization in xylene, the tissue sections were processed with a gradient of alcohol and subsequent phosphate-buffered saline (PBS) washing. The slides were immersed in H&E staining solutions according to the manufacturer’s instructions. Masson’s trichrome stain was performed to determine the deposition of the collagen fibers. The samples were stained by a Masson’s trichrome stain kit (Servicebio, Cat No. G1006-100ML), with hematoxylin, Biebrich scarlet-acid fuchsin, and aniline blue to color the collagen fibers selectively. The mean optical density (MOD) of each section was calculated using the software ImageJ (Version 2.14.0, National Institutes of Health, Bethesda, MD, USA).

#### 2.5.2. Tartrate-Resistant Acid Phosphatase (TRAP) Staining

The TRAP staining kit (Servicebio, Cat No. G1050-50T) was used to visualize osteoclasts. The TRAP working solution was meticulously prepared by adhering to the established protocols. The sections were subjected to a 2-h incubation at 37 °C in distilled water and then enveloped with the meticulously filtered TRAP working solution at 37 °C for 20–30 min. Hematoxylin was used for nuclear staining. The osteoclasts presented as multinucleated TRAP-positive cells with wine-red-stained cytoplasm. The number of cells was counted in three sections for each specimen.

#### 2.5.3. Immunohistochemical Staining

Immunohistochemical staining was performed using traditional methods described in our previous study [29]. Stomach enzyme (MXB Biotech, Fuzhou, China, Cat No. DIG-3009) was applied for antigen retrieval at 37 °C for 30 min, followed by overnight incubation with primary antibodies at 4 °C. Subsequent incubations included secondary antibodies, streptavidin–peroxidase, and freshly prepared DAB substrate (MXB Biotech, Cat No. DAB-0031). The following primary antibodies were used in this study: RANKL (1:1000; Abcam, Boston, MA, USA; Cat No. ab62516), osteoprotegerin (OPG, Toronto, ON, Canada; 1:200; Abcam; Cat No. ab73400), osteocalcin (OCN; 1:600; Abcam, Waltham, MA, USA; Cat No. ab93876), bone sialoprotein (BSP; 1:1000; CST, Danvers, MA, USA.; Cat No. 5648), osterix (OSX; 1:800; Abcam, Cambridge, MA, USA; Cat No. ab22552), interleukin (IL-1β (1:600; Abclonal, Woburn, MA, USA; Cat No. A16288), IL-6 (1:600; Proteintech, Sankt Leon-Rot, Germany; Cat No. 21865-1-AP), and TNF-α (1:600; Servicebio; Cat No. GB11188-100). For the semi-quantitative analysis, the MOD of each section was calculated using ImageJ (Version 2.14.0). The ROI was defined as the interproximal area between the first and second molars.

### 2.6. RNA Isolation and Quantitative Real-Time PCR (RT-PCR)

RNAiso (Servicebio, Cat No. G3019-100ML) was used to obtain total tissue or cellular RNA (n = 6/group), and reverse transcription was performed using the Prime Script RT Reagent Kit (TaKaRa, San Jose, CA, USA, Cat No. RR037A). The SYBR qPCR Master Mix (Vazyme, Nanjing, China, Cat No. Q311-02) was used for real-time PCR amplification of the cDNA on the Applied Biosystems QuantStudio 6 using the following thermocycling conditions: 95 °C for 30 s, 40 cycles at 95 °C for 10s, 62 °C for 34s, and 72 °C for 30s. The final gene expression results were calculated using the 2^−ΔΔCT^ method. Table S1 lists the primer sequences used in the present study.

### 2.7. Enzyme-Linked Immunosorbent Assay (ELISA) Analysis

Blood samples from the abdominal aorta were centrifuged at 3500× *g* for 5 min and stored at 4 °C (n = 3/group for each parameter). The supernatant was harvested and cryopreserved at −80 °C. The IL-1β, TNF-α, and IL-6 levels were detected using the corresponding ELISA detection kits (FANKEW, F2923-B, F3066-B, and F3056-B, respectively). Myeloperoxidase (MPO) activity was assessed by examining the neutrophils in the circulation using an ELISA kit (Solarbio, Beijing, China, SEKR-0073).

### 2.8. Cell Culture and Bacteria Culture

The mouse macrophage cell line RAW264.7 (third passage) was purchased from American Type Culture Collection, Manassas, WV, USA (ATCC^®^ TIB-71™). They were cultured in high-glucose Dulbecco’s modified Eagle medium supplemented with 10% fetal bovine serum (FBS) at 37 °C in 5% CO_2_ and 95% humidity. *P. gingivalis* (ATCC33277) was cultured anaerobically at 37 °C in trypticase soy broth supplemented with hemin, vitamin K1, and yeast extract. The concentration of *P. gingivalis* resuspension was measured according to the optical density at 600 nm (1 OD = 10^9^/mL). RAW264.7 cells were pre-incubated with or without 1 μg/mL CLH gel for 2 h prior to *P. gingivalis* stimulation. After replacing medium with fresh medium, the cells were infected with *P. gingivalis* (MOI = 100) for 6 h in each group.

### 2.9. Cytotoxicity Assay

The toxicity of CLH gel to macrophages was tested using a Cell Counting Kit-8 (CCK-8, Biosharp, Hefei, China, Cat No. BS350B). RAW264.7 cells were placed in 96-well plates at a density of 1 × 10^5^ cells per well and left overnight. After the cells attached, different concentrations of CLH gel (500, 200, 100, 10, 1, and 0 μg/mL) were added for 24, 48, and 72 h. Following the manufacturer’s instructions, 10 μL of CCK-8 dye and 90 μL of fresh serum-free medium were added to each well for 1 h at 37 °C. Absorbance was then measured at 450 nm using a microplate reader. Each experiment was repeated three times.

### 2.10. Western Blotting

The total protein from macrophages in cell culture was extracted using protease and phosphatase inhibitors, followed by SDS-PAGE and transfer to PVDF membranes. After blocking, the membranes were probed with antibodies against iNOS (Abclonal, Woburn, MA, USA, A3774, 1:1000), IL-1β (Abclonal, A16288, 1:1000), IRF8 (Affinity, Hong Kong, DF13627, 1:1000), STAT1 (Zenbio, Durham, NC, USA, R25799, 1:1000), SOCS1 (Zenbio, 340946, 1:1000), STAT2 (MCE, Singapore, YA057, 1:500), and β-actin (Proteintech, 66009-1-AP, 1:20,000), with β-actin serving as the loading control.

### 2.11. RNA Sequencing

Total RNA was extracted from cultured macrophages using TRIzol (TaKaRa, Cat No. 9019). Each condition was studied in three biological replicates. The samples were analyzed by Annoroad Gene Technology, Beijing, China, with 2 μg of RNA from each sample used for RNA preparation. Only samples with a RIN score greater than 7.0 and A260/A280 ratios between 1.7 and 2.1 were selected for sequencing. And we employed stringent data filtering to remove adapter contamination, low-quality reads, and reads with high N bases, ensuring mapping rates above 96%. After library inspection, different libraries were pooled based on concentration and target data volume. We utilized the MGI DNBSEQ-T7 platform for RNA sequencing, chosen for its high throughput and accuracy, which perfectly meets the requirements of our research. The sequencing depth was set to over 6 Gb per sample using paired-end sequencing (PE150), providing sufficient data for robust downstream analysis. This depth ensured comprehensive coverage to capture gene expression changes following cell stimulation. We performed gene set enrichment analysis (GSEA) using version 4.3.3 of the GSEA software. The gene sets used were from the Molecular Signatures Database (MSigDB), specifically the M2 collection (WikiPathways subset of CP). For the analysis, we used the GSEA software (v.4.3.3) to conduct gene set enrichment analysis with the Canonical Pathways gene sets downloaded from the MSigDB database. We set the following parameters for the gene set enrichment analysis: nperm = 1000, minSize = 15, maxSize = 500, permutation type = gene set.

### 2.12. Statistical Analysis

Based on previous literature, we adopted an alpha error of 0.05 and aimed for a power (1−β) of 90% to detect significant differences among the four groups [30]. To ensure sufficient power (90%) for detecting significant differences using ANOVA with four groups, we calculated a sample size of at least 8.4 rats per group, based on an alpha error of 0.05, variance of 10, and effect size of 5. Considering potential risks during modeling, we increased the number to 15 rats per group for reliable results. All data were statistically analyzed using the GraphPad Prism 10.0 software. Significant differences between groups were assessed using one-way analysis of variance (ANOVA) with Bonferroni correction, and a *p*-value of <0.05 indicated statistical significance.

## 3. Results

### 3.1. CLH Gel Suppressed Ligature-Induced Periodontal Tissue Destruction

**Figure 1:**
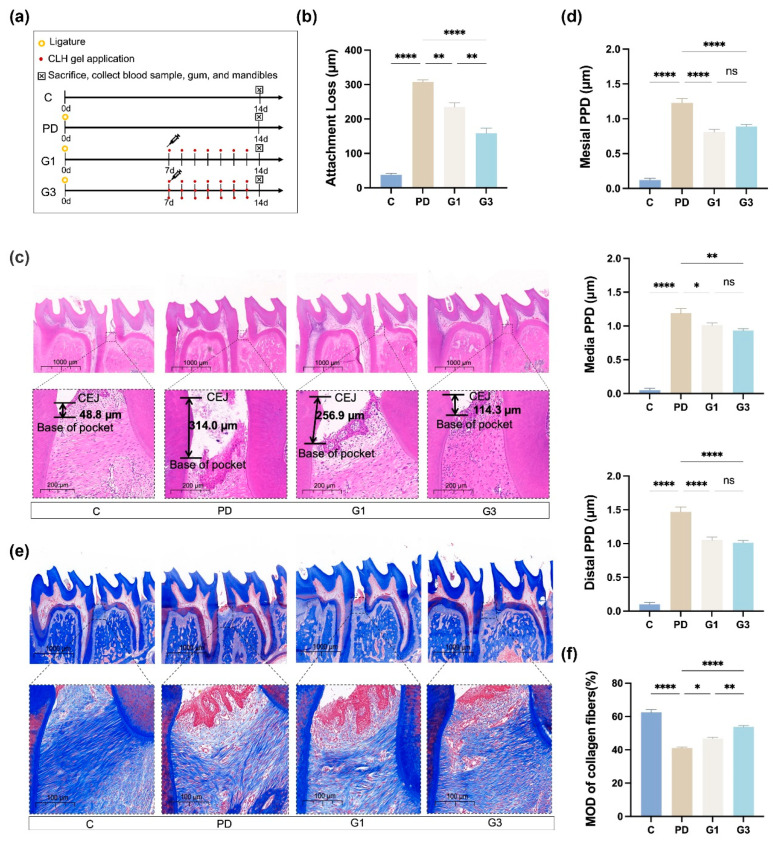
The experimental model and clinical parameters. Overview of the study schedule (**a**). C (no intervention), PD (ligature-induced periodontitis), G1 (ligature-induced periodontitis plus once-daily administration of CLH gel), G3 (ligature-induced periodontitis plus a thrice-daily administration of CLH gel). Average periodontal attachment loss (the distance between the cemento-enamel junction (CEJ) and the coronal position of the junctional epithelium) in the first molar (**b**). Results represent the means ± standard error of the mean (SEM) performed in four samples. H&E staining of the interdental epithelial attachment (black arrows indicate the actual epithelial attachment) between the first and second molars at low and high magnifications (**c**). Results represent the means ± SEM performed in four samples in each group. Clinical measurements of the PPD (distance from the gingival margin to the pocket bottom) at three sites (mesial, medial, distal) on the buccal side of the first molar (**d**). Results represent the means ± SEM of 10 samples. Immunohistochemical analysis of collagen fibers at low and high magnifications (**e**). Results represent the means ± SEM of collagen fibers of four samples in each group (**f**). Statistical analysis was performed with one-way ANOVA. ns, no significance. * *p* < 0.05; ** *p* < 0.01; **** *p* < 0.0001.

We used a well-established ligature-induced model to trigger periodontitis in rats, one of the most commonly used methods [31]. Silk sutures were tied around the mandibular first molars, causing mechanical irritation and facilitating bacterial accumulation, effectively inducing periodontitis within a short period. The modeling process is shown in Figure 1a. In the PD group, the silk threads were deeply embedded in the gingival sulcus of the first molars, along with substantial food debris. After removing the ligature, gingival edema, bleeding, and deep periodontal pockets were observed, indicating the successful induction of periodontitis, as described in previous studies [32,33]. No other clinical complications were observed during the study period.

Histopathology showed that the PD group had an average attachment loss of 307.06 ± 11.44 μm, while the G1 group had 234.73 ± 24.91 μm (*p* < 0.01) and the G3 group had 158.77 ± 30.09 μm (*p* < 0.0001), both significantly lower than the PD group (Figure 1b,c). Similar trends were observed for the PPDs from the mesial, medial, and distal sites (Figure 1d). The PPD on the distal was the deepest observed in the PD group, reaching up to 1.47 mm; however, the reductions in the G1 group at the three sites were not significantly different from those in the G3 group (Figure 1d). This suggests that in the rat periodontitis model, using the gel once or three times daily yields similar effects in terms of reducing probing depth on the 14th day.

Masson’s trichrome staining exhibited well-packed collagen in a parallel pattern in the control group, whereas loosely packed collagen fibers were observed in the PD group, suggesting severe destruction of the extracellular matrix (Figure 1e), as shown previously [34]. Increased amounts of ordered fibers were regenerated in the CLH-gel-treated group, and the fibers were 7% denser and richer in the G3 group than those in the G1 group (*p* < 0.01; Figure 1f).

### 3.2. CLH Gel Protected Against Ligature-Induced Alveolar Bone Loss

**Figure 2:**
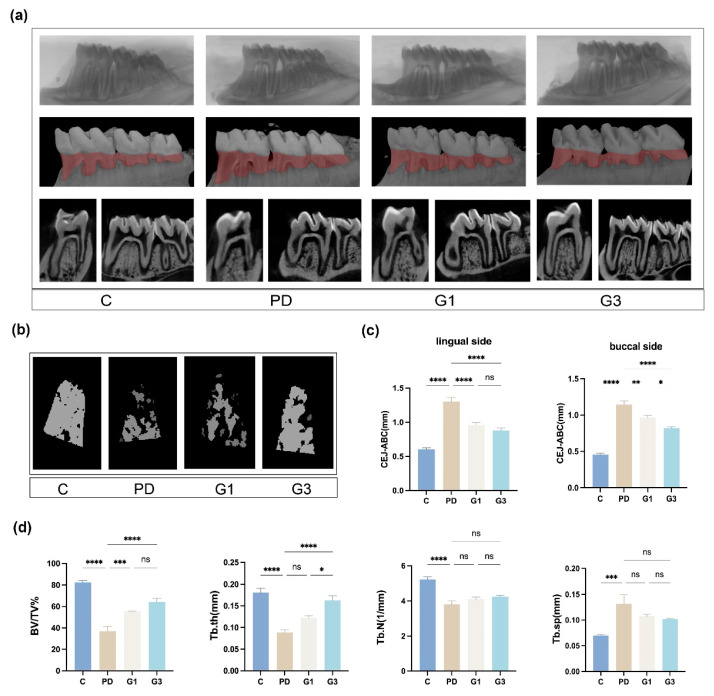
Alterations in the alveolar bone and the microarchitecture of the trabecular bone after modeling. Three-dimensional and bi-dimensional views of the mandibular molars from micro-CT scanning (**a**). Reconstructive images in red depict areas of bone loss. The reconstructive images of furcation areas in first molars (**b**). The distance between the CEJ and alveolar bone crest (ABC) on the buccal and lingual sides (**c**). Results represent the means ± SEM of 10 samples. Microarchitectural parameters of the alveolar bone (**d**). Bone volume to total tissue volume (BV/TV%), trabecular number (Tb.N), trabecular thickness (Tb.Th), trabecular separation (Tb.Sp). Results represent the means ± SEM of 10 samples. Statistical analysis was performed with one-way ANOVA. ns, no significance. * *p* < 0.05; ** *p* < 0.01; *** *p* < 0.001; **** *p* < 0.0001.

Compared to the control group, the rats in the PD group exhibited significant alveolar bone loss, with the CEJ-ABC distance increasing from 0.53 mm to 1.23 mm, BV/TV decreasing by 55.2% (*p* < 0.01), Tb.N decreasing from 5.23 mm^−1^ to 3.81 mm^−1^, Tb.Th decreasing from 0.18 mm to 0.09 mm, and Tb.Sp increasing from 0.07 mm to 0.13 mm. Local injection of the intrasulcular gel resulted in a decrease in bone absorption, as evident from the micro-CT with 3D reconstructions; the decrease was proportional to the frequency of gel administration (Figure 2a,b). The upregulated CEJ-ABC distances were partially reversed by CLH gel injection (Figure 2c).

Quantitatively, BV/TV was 18.42% higher in the G1 group and 27.42% higher in the G3 group than that in the PD group (Figure 2d). In the G3 group, the quantitative values of other parameters (Tb.Th, 0.16 mm; Tb.N, 4.24 mm^−1^; and Tb.Sp, 0.10 mm) were comparable to those in the control group (Tb.Th, 0.18 mm; Tb.N, 5.23 mm^−1^; and Tb.Sp, 0.07 mm; Figure 2d). These findings suggested that increasing the dosage of CLH gel led to a significant improvement in trabecular bone thickness (G1 vs. G3, *p* < 0.05).

### 3.3. CLH Gel Enhanced Osteogenic Differentiation and Reduced Osteoclastogenesis in Ligature-Induced Periodontitis

**Figure 3:**
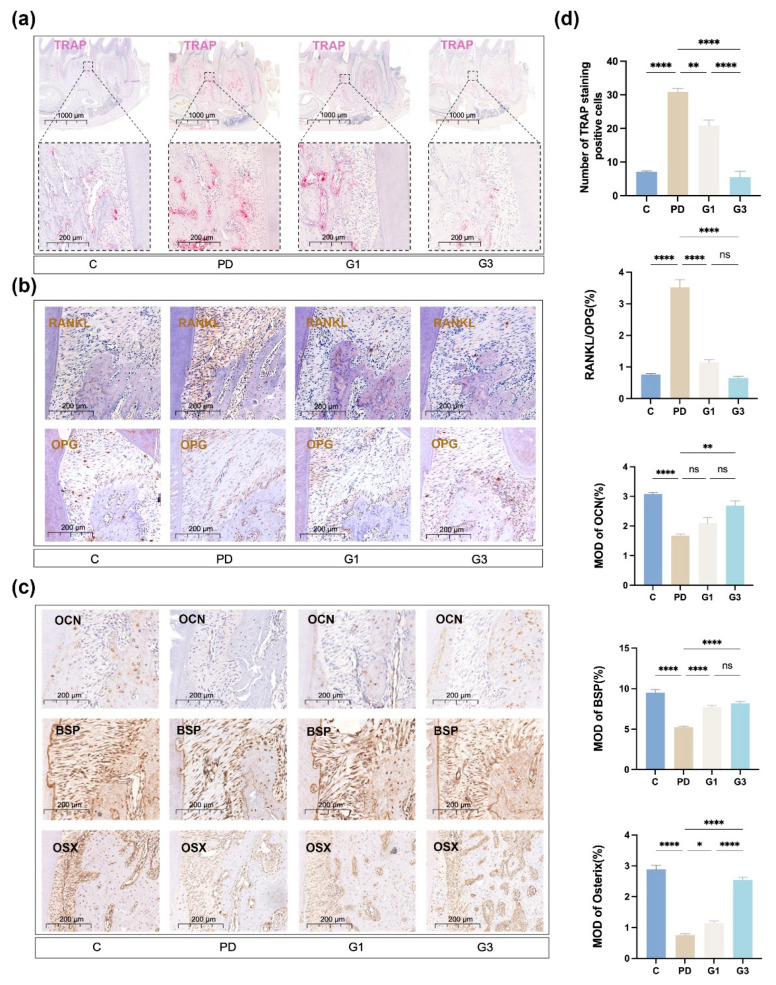
Representative histological observations and analyses of markers related to bone metabolism. TRAP staining of osteoclast infiltration in the periodontium, where osteoclasts are depicted in red, at both low and high magnifications (**a**). Immunohistochemical expression of OPG and RANKL in the interdental periodontal tissues (**b**). Immunohistochemical expression of OCN, BSP, and OSX in the interdental periodontal tissues (**c**). Immunohistochemical analysis (**d**). Means and standard deviations of the number of TRAP-positive cells, RANKL-positive cells/OPG-positive cells, OCN-positive cells, BSP-positive cells, and OSX-positive cells. Results represent the means ± SEM of four samples. Statistical analysis was performed with one-way ANOVA. ns, no significance. * *p* < 0.05; ** *p* < 0.01; **** *p* < 0.0001.

TRAP staining was performed to evaluate the distribution and number of multinucleated osteoclasts in ROI (Figure 3a). The number of giant and hyper-nucleated osteoclasts was significantly increased in the PD group (*p* < 0.0001) and markedly reduced in the G1 (*p* < 0.001) and G3 (*p* < 0.0001) groups in a dose-dependent manner (G1 vs. G3, *p* < 0.0001) (Figure 3d). The ratio of RANKL to OPG was significantly increased in the PD group (*p* < 0.0001), indicating that ligature placement exacerbated the bone destruction process. CLH gel treatment reduced the RANKL/OPG ratio from 3.52 to 1.15 (G1) and 0.65 (G3) (Figure 3b,d). Gel treatment once a day inhibited the RANKL/OPG ratio by 34%, whereas three treatments a day resulted in a more significant reduction (57%) compared to the PD group.

As shown in Figure 3c, the periodontitis group exhibited a decrease in the expression of the osteogenic markers (OCN, BSP, and OSX) compared to those in the control group; alternatively, the CLH gel effectively increased the expression levels of these proteins in the interdental areas between the first and second molar. The G3 group demonstrated more evident OCN and OSX expression than the G1 group (Figure 3d).

### 3.4. CLH Gel Attenuated Ligature-Induced Inflammation in Gingival Tissues

**Figure 4:**
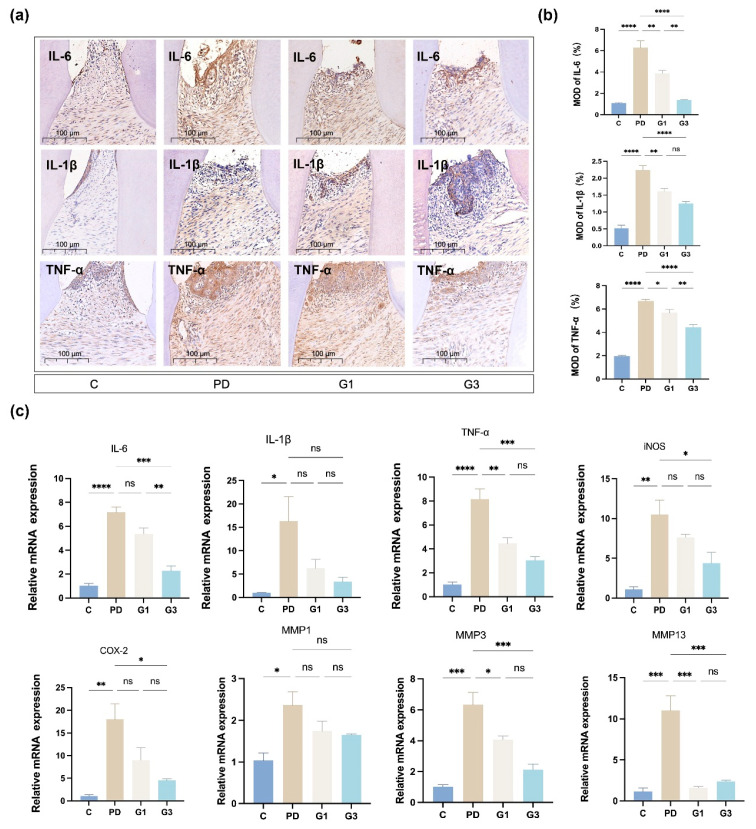
Effects of experimental periodontitis on the inflammatory status of the gingival tissues. Immunohistochemical expression of IL-6, IL-1β, and TNF-α in the interdental gingiva and periodontal ligament (**a**). Immunohistochemical analysis of IL-6, IL-1β, and TNF-α (**b**). Means and standard deviations of the number of positive cells. Results represent the means ± SEM of four samples. The expression of inflammation-related genes in rat gingival tissues (**c**). Total RNA was extracted from the gingival tissue around the first molars, and the mRNA levels of *TNF-α*, *IL-6*, *IL-1β*, *iNOS*, *COX2*, *MMP-1*, *MMP-3*, and *MMP-13* were determined by qRT-PCR. Results represent the means ± SEM of three samples. Statistical analysis was performed with one-way ANOVA. ns, no significance. * *p* < 0.05; ** *p* < 0.01; *** *p* < 0.001; **** *p* < 0.0001.

We further evaluated the inflammatory response by measuring the expression of TNF-α, IL-6, and IL-1β at protein (Figure 4a,b) and mRNA (Figure 4c) levels in the gingival component. Ligation induced a substantial elevation of all the examined proinflammatory cytokines, inducible nitric oxide synthase (iNOS) (*p* < 0.01), cyclooxygenase-2 (COX2) (*p* < 0.01), TNF-α (*p* < 0.0001), IL-6 (*p* < 0.0001), and IL-1β (*p* < 0.05). And treatment with CLH gel exhibited a quantitative reduction in the levels of TNF-α (G1 vs. PD, *p* < 0.01; G3 vs. PD, *p* < 0.01) and IL-6 (G3 vs. PD, *p* < 0.001) (Figure 4c). The mRNA level of *IL-6* decreased with the frequency of gel administration (G1 vs. G3, *p* < 0.01). In addition, administering CLH gel three times a day significantly decreased the mRNA levels of *iNOS*, *COX2*, *TNF-α*, *IL-6*, and *IL-1β* in gingival tissues by 3.99, 2.39, 2.67, 3.15, and 4.82 times, respectively (Figure 4c). In the current study, the ligature also induced a significant upregulation of *MMP-1* (*p* < 0.05), *MMP-3* (*p* < 0.001), and *MMP-13* (*p* < 0.001), with increases of 2.29, 6.22, and 9.62 times the control, respectively. The CLH gel treatment (once and three times a day) significantly (*p* < 0.05) reduced the mRNA expression levels of *MMP-3* and *MMP-13* (Figure 4c).

### 3.5. CLH Gel Curbed Ligature-Induced Accumulation of Inflammatory Cells and the Secretion of Cytokines in Circulation

**Figure 5:**
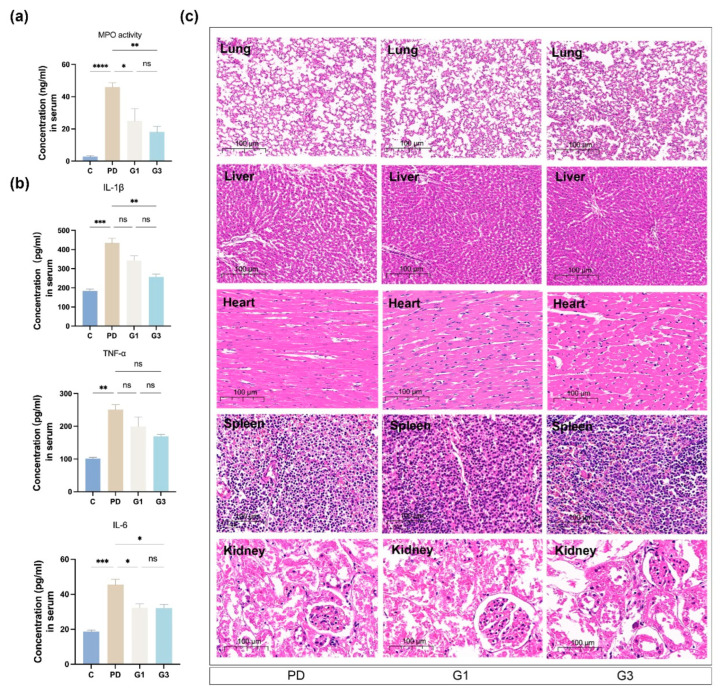
Effects of experimental periodontitis on the inflammatory status of the serum and systemic organs. Myeloperoxidase (MPO) activity in the serum of rats (**a**). Results represent the means ± SEM of three samples. Inflammatory mediator production (TNF-α, IL-6, IL-1β) in the serum of the rats (**b**). ELISA kits were employed to detect the protein levels of the inflammatory cytokines. Results represent the means ± SEM of three samples. H&E staining of major organs (heart, liver, spleen, lung, and kidney) in experimental rats (**c**). No obvious damage was observed in the visceral organs (n = 6/group). Statistical analysis was performed with one-way ANOVA. ns, no significance. * *p* < 0.05; ** *p* < 0.01; *** *p* < 0.001; **** *p* < 0.0001.

MPO is predominantly present in the azurophilic granules of neutrophils and is used as a quantitative marker of neutrophil infiltration [35]. In the PD group, a significant increase in MPO activity (46.05 ng/mL) was seen in the serum (Figure 5a). The serum levels of IL-6 (18.70 pg/mL in C vs. 45.49 pg/mL in PD, *p* < 0.001), IL-1β (183.43 pg/mL in control vs. 434.24 pg/mL in PD, *p* < 0.0001), and TNF-α (101.73 pg/mL in control vs. 250.80 pg/mL in PD, *p* < 0.01) were significantly higher in the PD group compared to the control group (Figure 5b), indicating systemic inflammation. The local administration of CLH gel in the sulcus effectively reduced the concentrations of MPO and the inflammatory factors in the blood. Notably, the gel’s anti-inflammatory effect was most pronounced in the G3 group, with reduced concentrations of IL-6 (32.13 pg/mL), IL-1β (256.82 pg/mL), and TNF-α (169.73 pg/mL), with significant statistical differences observed for IL-1β (*p* < 0.01) and IL-6 (*p* < 0.05) compared to the PD group.

Histopathological analyses of the heart, liver, spleen, lung, and kidney revealed no apparent parenchymal organ damage in all the groups, with no significant differences among the PD, G1, and G3 groups (Figure 5c).

### 3.6. CLH Gel Inhibited the Transformation of Macrophages into an Inflammatory Phenotype Under Stimulation by P. gingivalis

**Figure 6:**
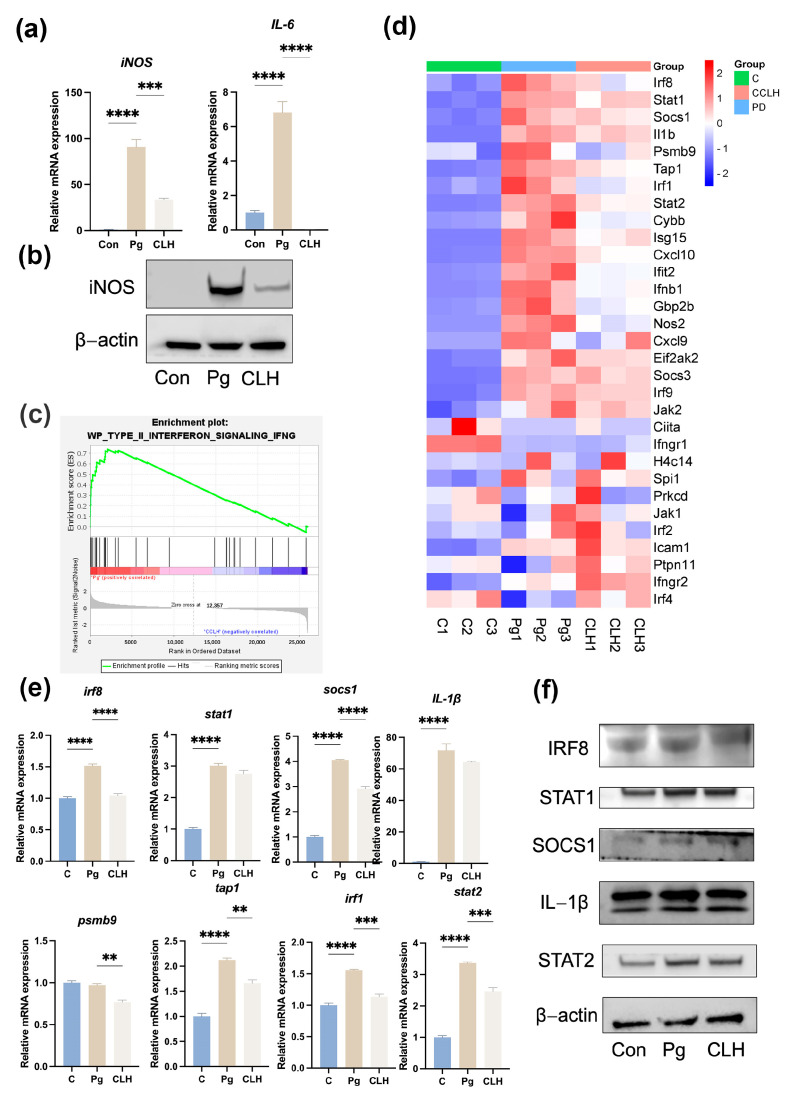
Anti-inflammatory effects of CLH gel on *P. gingivalis*-infected RAW264.7 cells. (**a**) iNOS and IL-1β mRNA levels in RAW264.7 cells after *P. gingivalis* (MOI = 100) stimulation for 6 h, with 1 µg/mL CLH treatment. (**b**) iNOS protein expression under the same conditions. (**c**) Gene set enrichment analysis (GSEA) line graph of functional gene set enrichment scores. (**d**) Heatmap of differentially expressed genes in the Type II interferon network, ranked by enrichment score. (**e**) mRNA levels of irf8, stat2, socs1, psmb9, tap1, irf1, IL-1β, and stat1 under the same conditions. (**f**) Protein expressions of IRF8, STAT2, SOCS1, IRF1, IL-1β, and STAT1 in RAW264.7 cells after stimulation with *P. gingivalis* combined with 1 µg/mL CLH for 6 h. with 1 µg/mL CLH. Statistical analysis was performed with one-way ANOVA. ns, no significance. ** *p* < 0.01; *** *p* < 0.001; **** *p* < 0.0001.

To further explore the molecular mechanisms by which the gel combats periodontal infection, we conducted in vitro experiments using a *P. gingivalis*-infected mouse bone-marrow-derived macrophage model, which allowed us to assess the gel’s effects on immune cell function. To ensure that the effects observed were due to the pharmacological action of CLH gel and not cytotoxicity, we first conducted a CCK-8 experiment, confirming that CLH gel below 10 µg/mL did not affect macrophage proliferation and viability (as shown in Figure S1). Then, RAW264.7 cells were pre-incubated with 1 µg/mL CLH gel for 2 h. After changing to fresh medium, live *P. gingivalis* was added and co-cultured for 6 h. Under bacterial stimulation, macrophages exhibited a significant increase in inflammatory markers, with IL-6 mRNA levels upregulated by 85.5-fold (*p* < 0.0001) and iNOS mRNA levels increasing by 6.75-fold (*p* < 0.0001), indicating a clear trend toward inflammatory polarization, as shown in Figure 6a,b. However, 1 µg/mL CLH gel significantly reduced IL-6 mRNA levels (*p* < 0.0001) and iNOS mRNA levels (*p* < 0.001), demonstrating the effectiveness of CLH gel in counteracting macrophage inflammation, which is consistent with the trend at the protein level. The reduced expression of inflammatory cytokines in periodontal tissues and macrophages suggested that the gel can inhibit the release of inflammatory cytokines by immune cells in periodontitis.

### 3.7. CLH Gel Reduced Macrophage Inflammation in Periodontitis by Modulating Type II Interferon Networks

Using the same cell culture method as above, RNA was collected from the samples and subjected to RNA sequencing. The results showed significant differences in genes related to the Type II interferon networks among the three groups, as shown in Figure 6c,d. We used PCR and Western blot to confirm the expression of the most significantly changed genes and proteins indicated by the GSEA. The results showed that markers in Type II interferon networks, such as IRF8, STAT2, SOCS1, IRF1, IL-1β, and STAT1, were involved in the macrophage response to live *P. gingivalis* infection. CLH gel effectively reduced macrophage inflammation by improving the changes in these markers at both the protein and mRNA levels (Figure 6e,f). Specifically, our PCR results confirmed that when treated with CLH gel, the mRNA levels of irf8, socs1, psmb9, tap1, irf1, and stat2 were downregulated 1.45-fold (*p* < 0.0001), 1.39-fold (*p* < 0.0001), 1.26-fold (*p* < 0.01), 1.28-fold (*p* < 0.01), 1.37-fold (*p* < 0.001), and 1.37-fold (*p* < 0.01), respectively. This highlights the importance of Type II interferon networks in CLH’s action against periodontal pathogens, potentially linking these networks to bone metabolism changes seen in vivo.

## 4. Discussion

In this study, we initially validated the therapeutic efficacy of CLH gel in an in vivo rat model of periodontitis, demonstrating its ability to attenuate inflammation in both periodontal tissues and systemic circulation. Additionally, CLH gel reduced alveolar bone resorption by inhibiting osteoclast activity and promoting osteogenic processes. To further elucidate the underlying mechanisms influencing inflammation and bone metabolism, we conducted in vitro experiments using a *P. gingivalis*-stimulated macrophage model. These findings revealed that CLH gel mediates its anti-inflammatory effects through the regulation of Type II interferon networks and macrophage polarization. Collectively, the in vivo and in vitro results support the hypothesis that CLH gel exerts beneficial effects in periodontitis by modulating inflammatory responses and bone metabolism.

The pharmacological potential of different species within the daisy family, such as *Matricaria chamomilla* and Roman chamomile, suggests that this botanical family holds promise as a valuable source of novel bioactive compounds [16]. Chamomile is commercially available for medicinal use in various forms, including capsules, tablets, tinctures, mouthwash, and lotions [16]. Recently, CLH gel has been used in eczema and atopic dermatitis due to its anti-inflammatory and soothing properties. Researchers also took advantage of these properties to treat oral ulcers and mucositis [36]. And a previous study had reported that the CLH gel might have comparable benefits to minocycline hydrochloride ointment in improving the clinical parameters in patients [23]. Regarding the impact of two different dosing frequencies on clinical indicators of periodontitis, the lack of significant differences between the three-times-daily and once-daily groups suggests that frequent dosing may not be necessary in the short term. This contrasts with treatment recommendations for mucosal ulcers, as stated in the product instructions, where increased dosing is aimed at enhancing pain relief. However, at the molecular level, this study further demonstrated the gel’s effectiveness in reducing inflammation and promoting bone formation, particularly in rats treated three times daily.

CLH gel demonstrated remarkable anti-inflammatory and immunomodulatory functions. Activated macrophages phagocytize pathogens and secrete proinflammatory signals, and changes in phenotype and function are crucial pathways affecting tissue damage and repair [37]. In periodontitis, activated macrophages phagocytize invading pathogens and secrete proinflammatory signals, further recruiting T lymphocytes and neutrophils to enhance innate immune defenses against periodontal pathogens [38,39,40,41]. In this study, *P. gingivalis* stimulated macrophages to adopt an inflammatory phenotype. In animal experiments, elevated MPO activity and increased levels of TNF-α, IL-1β, IL-6, iNOS, and COX-2 confirmed the inflammatory response. Notably, 1 µg/mL CLH gel effectively inhibited macrophage inflammation in vitro and, in vivo, significantly reduced inflammation.

Additionally, our study confirmed the gel exerts a strong inhibitory effect on MMP activity, which plays a crucial role in destruction of periodontal structures [42,43,44]. Numerous studies have shown that elevated MMP levels, particularly MMP-1, MMP-3, and MMP-13, contribute to collagen breakdown, bone resorption, and the intensification of inflammatory responses in periodontitis, consistent with their well-documented role in degrading key structural components like Type I, III, IV, and IX collagen, fibronectin, laminin, and proteoglycans [45,46,47]. MMP-13, in particular, is strongly linked to bone loss, while MMP-3 is associated with overall matrix degradation. However, the application of CLH gel, both once daily and thrice daily, effectively reduced the expression of MMP-3 and MMP-13. The potent inhibitory effect on MMPs may be linked to the anti-inflammatory properties of chamomile, a key component of the gel, known for suppressing inflammatory mediators and potentially inhibiting the NF-κB pathway. Since NF-κB activation drives MMP expression, this suppression likely reduces the inflammatory cascade that exacerbates tissue damage and impairs healing processes [48]. These findings suggest that CLH gel holds promise as an effective therapeutic agent in managing periodontitis by targeting both inflammation and tissue degradation mechanisms.

Our study is the first to demonstrate that CLH gel modulates bone metabolism. Some key osteogenic markers (OCN, BSP, and OSX) were recovered in the CLH-treated group, underscoring the gel’s dual role in improving bone microstructure, providing a foundation for its therapeutic potential in periodontitis. The molecular mechanisms underlying the beneficial effects of CLH gel on bone metabolism are multifactorial. In the periodontitis group, aside from the decrease in osteogenic markers, we also observed that bone resorption was associated with elevated RANKL. RANKL, a key regulator of osteoclastogenesis, facilitates the differentiation and activation of osteoclasts by binding to its receptor, RANK, on osteoclast precursors [49]. OPG, acting as a decoy receptor for RANKL, inhibits this process by blocking the RANK–RANKL interaction, thereby reducing osteoclast formation. By focusing on the RANKL/OPG balance, our study provides further evidence of CLH gel’s potential to not only reduce inflammation but also reduce its ratio in bone metabolism. One possible explanation is the inhibitory effect of chamomile on this axis, as previously reported, supporting the hypothesis that chamomile may effectively stimulate osteoblast differentiation [50,51] and suppress osteoclastogenesis via the RANKL/OPG axis [52]. Additionally, our findings indicate that the Type II interferon (IFN-γ) pathway is closely tied to the RANKL axis, particularly through macrophage polarization. IFN-γ plays an important biological role in the body. Its impact on osteoclasts depends on IFN-γ levels, RANKL, and the stage of osteoclast differentiation [53]. In chronic inflammatory conditions, it drives M1 polarization, which heightens inflammation and RANKL/OPG-axis-induced bone loss by releasing cytokines like IL-1β and TNF-α [54]. Our in vitro data align with this, showing IFN-γ pathway activation (IRF8, STAT1, IL-1β) during macrophage polarization under *P. gingivalis* infection, suggesting a positive link between RANKL and IFN-γ in regulating osteoclast differentiation in periodontitis. Interestingly, other studies report that early RANKL exposure in bone marrow macrophages might induce resistance to IFN-γ, reducing osteoclast formation [54,55], which highlights another layer of interaction between IFN-γ and the RANKL/OPG axis. Further research is essential to clarify how CLH gel affects these pathways.

Based on our findings, we have confirmed for the first time that CLH gel effectively treats periodontitis in rats by reducing inflammation and bone loss. The anti-inflammatory mechanism likely involves regulating Type II interferon networks, affecting macrophage-mediated inflammation, neutrophil accumulation, and the balance between osteoblast and osteoclast activity (graphical abstract). Increasing the application frequency from once to three times daily enhanced the gel’s efficacy against inflammation and tissue damage without causing biocompatibility or safety issues.

Despite the strengths of this study, there are certain limitations. Animal models may not fully capture the complexity of human periodontitis. The ligature-induced model, while widely used for its ability to rapidly provoke severe bone loss, is highly technique-sensitive [26,56]. Excessive force during ligature placement can injure gingival tissues and cause bleeding, potentially leading to additional periodontal damage in the model group. Nevertheless, the significant recovery observed in the treatment group under the same conditions highlights the potential therapeutic efficacy of CLH gel. Moreover, the duration of gel application in this study was limited, and its long-term systemic toxicity remains undetermined. In addition, the in vitro experiments in this study only verified the response of macrophages to the gel under periodontal pathogen infection, but the complex interactions between other immune cells in the periodontal microenvironment were not fully reflected. Therefore, further studies are required to explore the underlying mechanisms of its regulatory effects on other cells. Large-scale clinical studies are essential to validate the safety, long-term efficacy, and optimal dosing of CLH gel as an adjunctive therapy in periodontitis management alongside mechanical plaque removal.

## 5. Conclusions

This study highlights the therapeutic potential of CLH gel in mitigating the progression of periodontitis. The gel exerts its anti-inflammatory effects by modulating macrophage polarization via the Type II interferon pathway under *P. gingivalis* infection, while also regulating bone resorption by balancing the RANKL/OPG axis and enhancing osteogenesis in a rat periodontitis model. Specifically, CLH gel reduced proinflammatory cytokines such as TNF-α and IL-6 in both local gingival tissues and serum, while increasing the expression of osteogenic markers like OCN and OSX. These findings suggest that CLH gel may serve as an adjunct to conventional non-surgical periodontal therapy, such as scaling and root planing, by offering anti-inflammatory and tissue-regenerative benefits. These benefits may lead to more effective management of periodontal disease, enhanced patient comfort, improved long-term health of periodontal tissues and tooth stability, a higher overall standard of oral healthcare, and an improved quality of life.

## Data Availability

The data that support the findings of this study are available on request from the corresponding author. The RNA-seq data in this study can be found in online repositories. The names of the repository/repositories and accession number(s) can be found below: https://www.ncbi.nlm.nih.gov/, PRJNA1172147.

## Supplementary Materials

The following supporting information can be downloaded at: https://www.mdpi.com/article/10.3390/biomedicines12112629/s1, Figure S1: Toxicity of CLH gel to macrophages; Table S1: Primers used in RT-PCR.
