# Supplementary material for: Chamomile Tincture and Lidocaine Hydrochloride Gel Ameliorates Periodontitis: A Preclinical Study
Source: Biomedicines. 2024 Nov 17;12(11):2629. doi: 10.3390/biomedicines12112629 (PMC11592006; doi:10.3390/biomedicines12112629)
Supplement: Supplementary file 1 [file biomedicines-12-02629-s001.zip › biomedicines-3178758-supplementary.pdf]

Table S1. Primers used in RT-PCR.

Target	Forward Sequence (5'-3')	Reverse Sequence (5'-3')
TNF-α(human)	TTCCCAAATGGGCTCCCTCT	GTGGGCTACGGGCTTGTCAC
IL-1β(human)	TGTGATGTTCCCATTAGAC	AATACCACTTGTTGGCTTA
IL-6(human)	TGCCTTCTTGGGACTGAT	TTGCCATTGCACAACTCT
iNOS(human)	CCTGGTGCAAGGGATCTTGG	GAGGGCTTGCCTGAGTGAGC
COX-2(human)	CTCAGCCATGCAGCAAATCC	GGGTGGGCTTCAGCAGTAAT
MMP1(human)	GACGTGACCGACAACAGGTGA	GGGGAACATTAGTGCTCCTACATC
MMP3(human)	TCCCTGTTCAGCCATCCCTTG	TCGCTCTGGTAGCCCTTCTC
MMP13(human)	TGTTTGCAGAGCACTACTTGAA	CAGTCACCTCTAAGCCAAAGAAA
GAPDH(human)	GGTGGACCTCATGGCCTACAT	GCCTCTCTCTTGCTCTCAGTATCCT
iNOS(mouse)	CAGGGAGAACAGTACATGAACAC	TTGGATACACTGCTACAGGGA
IL-6(mouse)	CCACTTCACAAGTCGGAGGCTTA	CCAGTTTGGTAGCATCCATCATTTC
irf8(mouse)	CGGGGCTGATCTGGGAAAAT	CACAGCGTAACCTCGTCTTC
stat1(mouse)	GCTGCCTATGATGTCTCGTTT	TGCTTTTCCGTATGTTGTGCT
socs1(mouse)	CTGCGGCTTCTATTGGGGAC	AAAAGGCAGTCGAAGGTCTCG
psmb9(mouse)	CATGAACCGAGATGGCTCTAGT	TCATCGTAGAATTTTGGCAGCTC
tap1(mouse)	CTTGGATGATGCCACCAGTG	AGAAGAACCGTCCGAGAAGC
irf1(mouse)	ATGCCAATCACTCGAATGCG	TTGTATCGGCCTGTGTGAATG
stat2(mouse)	GTTACACCAGGTCTACTCACAGA	TGGTCTTCAATCCAGGTAGCC
IL-1β(mouse)	TTCAGGCAGGCAGTATCACTC	GAAGGTCCACGGGAAAGACAC
β-actin(mouse)	GATTACTGCTCTGGCTCCT	TGGAAGGTGGACAGTGAG

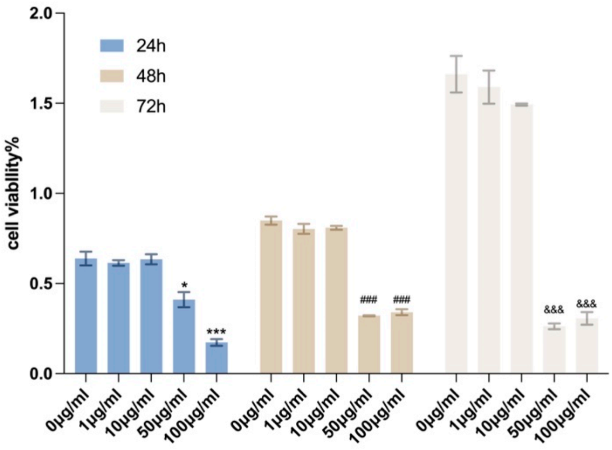

Figure S1 Toxicity of CLH gel to macrophages.

CCK-8 test of cell viability of RAW264.7 with CLH gel concentration ranging from 100, 50, 10, 1 to 0 μg/ml. Statistical analysis was performed with one-way ANOVA. ns, no significance. *(compared with 0μg/ml at 24h)or # (compared with 0μg/ml at 48h)or & (compared with 0μg/ml at 72h), $p < 0.05$; ***or ### or &&&, $p < 0.001$.
